# Comparison of the Filtration Efficiency of Different Face Masks Against Aerosols

**DOI:** 10.3389/fmed.2021.654317

**Published:** 2021-07-02

**Authors:** Connor Stahl, Kevin Frederick, Sachin Chaudhary, Christopher J. Morton, Douglas Loy, Krishna Muralidharan, Armin Sorooshian, Sairam Parthasarathy

**Affiliations:** ^1^Department of Chemical and Environmental Engineering, University of Arizona, Tucson, AZ, United States; ^2^Department of Materials Science and Engineering, University of Arizona, Tucson, AZ, United States; ^3^Division of Pulmonary, Allergy, Critical Care and Sleep Medicine, University of Arizona, Tucson, AZ, United States; ^4^University of Arizona Health Sciences Center for Sleep and Circadian Sciences, University of Arizona, Tucson, AZ, United States; ^5^Department of Hydrology and Atmospheric Sciences, University of Arizona, Tucson, AZ, United States

**Keywords:** COVID-19, mask, aerosol, SARS-CoV-2, viral transmission

## Abstract

**Background:** The severe acute respiratory syndrome coronavirus 2 (SARS-CoV-2) pandemic can spread through virus-containing aerosols ( ≤ 5 μm) and larger airborne droplets. Quantifying filtration efficiency of different kinds of masks and linings for aerosols that fall within the most penetrating particle size (80-400 nm) is critical to limiting viral transmission. The objective of our experiment was to compare the “real-world” filtering efficiency of different face masks for fine aerosols (350 nm) in laboratory simulations.

**Methods:** We performed a simulated bench test that measured the filtering efficiency of N95 vs. N99 masks with elastomeric lining in relation to baseline (“background”) aerosol generation. A mannequin head was placed within a chamber and was attached to an artificial lung simulator. Particles of known size (350 ± 6 nm aerodynamic diameter) were aerosolized into the chamber while simulating breathing at physiological settings of tidal volume, respiratory rate, and airflow. Particle counts were measured between the mannequin head and the lung simulator at the tracheal airway location.

**Results:** Baseline particle counts without a filter (background) were 2,935 ± 555 (SD) cm^−3^, while the N95 (1348 ± 92 cm^−3^) and N99 mask with elastomeric lining (279 ± 164 cm^−3^; *p* <0.0001) exhibit lower counts due to filtration.

**Conclusion:** The filtration efficiency of the N95 (54.1%) and N99 (90.5%) masks were lower than the filtration efficiency rating. N99 masks with elastomeric lining exhibit greater filtration efficiency than N95 masks without elastomeric lining and may be preferred to contain the spread of SARS-CoV-2 infection.

## Introduction

The severe acute respiratory syndrome coronavirus 2 (SARS-CoV-2) pandemic continues to spread worldwide and has caused almost 2.6 million deaths as of March 11, 2021 (1). Research suggests that such spread is mediated by airborne transmission and that face masks are more effective in limiting the spread of SARS-CoV-2 infection compared to social distancing (2). Considering that there are various mask types, inter-disciplinary research comparing mask filtration efficacy are needed (2). Large airborne particles (>5 μm) are more likely to deposit in the head airways, whereas smaller particles are likely to deposit in the conducting airways of the lungs. Fine aerosols (0.01-1 μm) in single breath assessments are associated with least deposition (~10%), but under multiple breath conditions they can continue to be retained within the lung and deposit to a greater degree through gravitational forces (3, 4). Therefore, prolonged exposure to 100-500 nm particles can lead to progressively greater cumulative deposition of virus inoculum if the exposure time is sufficiently long. Interestingly, such a particle size corresponds to the 300 nm particulate size threshold used for grading the filtration efficiency of masks (5) and the most penetrating particle size of single-filter masks (6). Moreover, in field tests, investigators have reported poor performance of N95 masks (7). A key reason for such a finding may have been poor mask seal afforded by N95 masks which could be prevented by elastomeric linings. Accordingly, the overarching objective of our experiment was to compare the “real-world” filtration efficiency of different face masks for fine aerosols over multiple breaths and to study the effect of elastomeric lining in laboratory simulations.

## Methods

**Figure 1:**
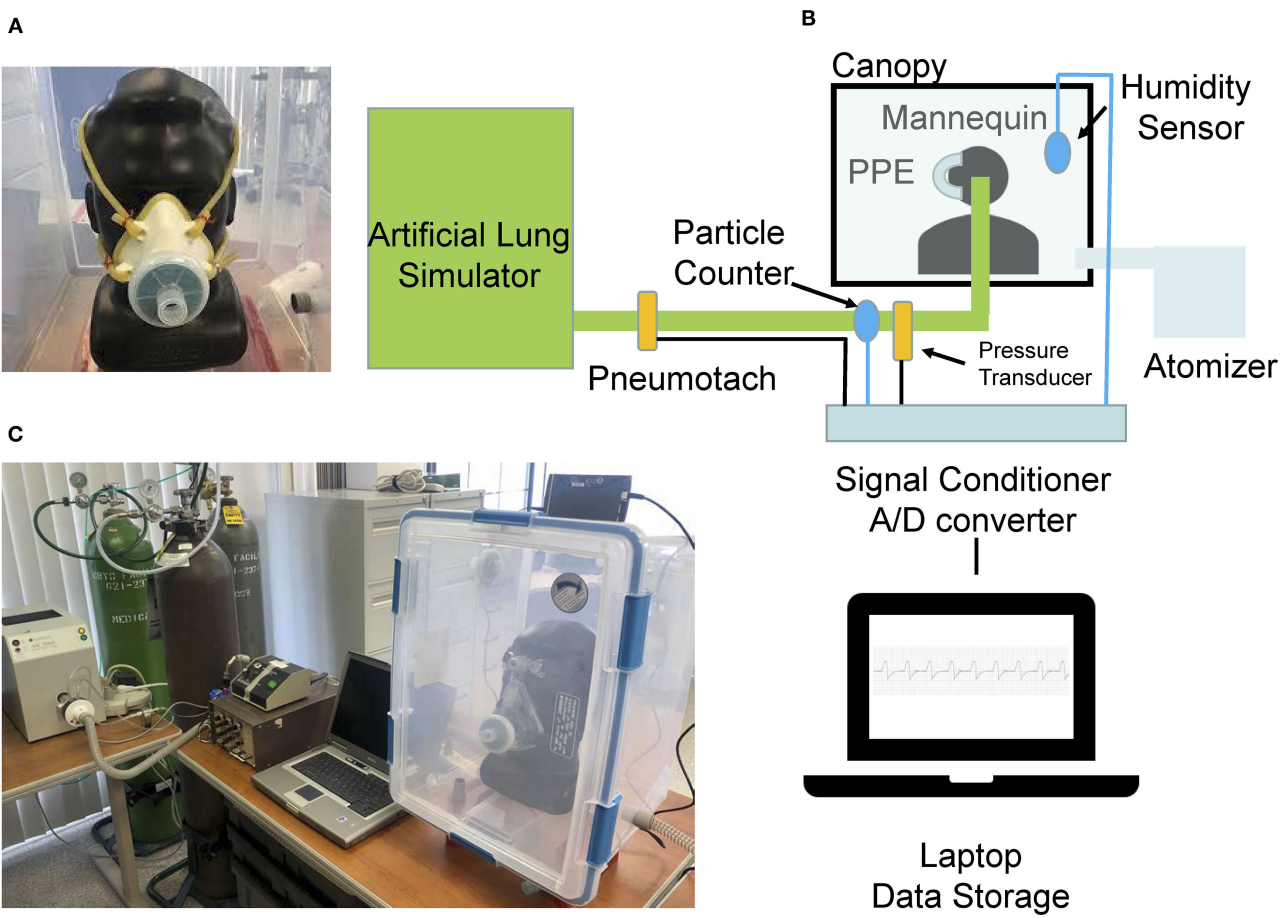
Images of the **(A)** N99 mask with elastomeric lining and **(B)** bench experiment set-up. **(C)** A picture of the experiment set-up. Image of a N95 respiratory that was snugly fit on mannequin head.

**Figure 2:**
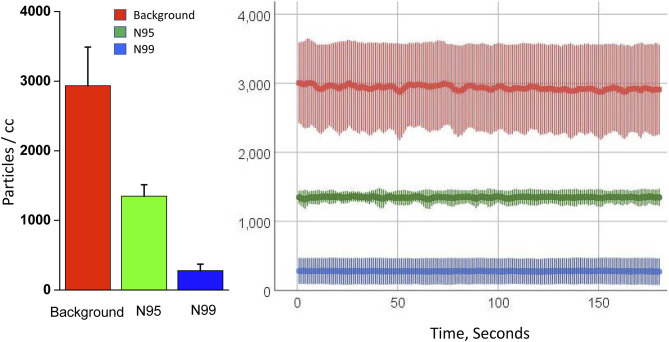
Mean and standard deviation of data for the last 3-min are shown on the panel for the background condition (no mask) and the N95 and N99 masks.

We performed a bench test of a N95 filtering-facepiece respirator (N95 respirator, N95;1860S; 3M Company, St. Paul, MN) and a 3D printed full face mask with elastomeric lining with a N99 filter, or no mask (baseline) using an artificial lung simulator (ASL-5000; IngMar Medical, Inc. Pittsburgh, PA; Figures 1, 2) (8). The masks were applied to a mannequin head placed inside a chamber into which aerosol could be introduced. The N95 mask and N99 mask were fitted as well as possible by ensuring the nasal bridge wire was contoured to the face and that the straps were tight to provide adequate pressure to the face to provide the best fit possible (Figures 1, 2). The mannequin head was connected through an artificial passageway (mimicking the tracheal airway) to the artificial lung simulator. A constant-rate atomizer with controllable liquid supply flow rate and an in-line desiccant dryer (all stainless-steel construction) (Brechtel Manufacturing Inc. Model 9200) were used for aerosol generation. Polystyrene latex sphere (PSL; Thermo Scientific 3000 Series Nanosphere) aerosols were generated at a fixed diameter of 350 ± 6 nm. The generated aerosol sample was transported to a sealed chamber (93.4 L) containing the mannequin head and allowed to equilibrate prior to data collection. Particle number concentration was measured within the tracheal airway at 1 Hz resolution using a butanol-based mixing-type condensation particle counter (Brechtel Manufacturing Inc. Model 1710). Measurements were collected using the instrument manufacturer's MCPC_Recording_V2.1 software. Proximal to the artificial lung simulator, measurements of flow (heated pneumotachograph, Fleisch, Lausanne, Switzerland) and airway pressure (Validyne, Northridge, CA) were made and stored in a laptop for subsequent review and analysis (Figure 1).

Fine aerosol particles with a diameter of 350 ± 6 nm were assessed because such aerosols are capable of spreading the virus at short to medium distances (several meters or entire room) as they remain suspended in the air (9, 10). We chose to affix the masks on mannequin heads to more closely mimic real-life scenarios rather than merely test the filters to address the potential for leakage or penetration of the interface between the mask and the face. The N95 mask has built in metal strips that allow affixing the mask on the face of the mannequin whereas the N99 mask was 3-D printed with an elastomeric lining that can afford a better fit to the mannequin face. Three runs of 15 min each were performed for each of the two masks and at baseline without a mask and the particle counts were measured over the last 3-min period when there were stable particle concentrations. Particles were measured in 3-min blocks at 1 s time resolution after particle generation and the chamber equilibrated for each mask test. This gave sufficient data for each respective mask (180 points per mask or 540 points per group) allowing for robust comparison of the masks as well as the variation from inhalation and exhalation through the masks. The respiratory rate (12 breaths per minute), tidal volume (500 ml), peak inspiratory flow rate (60 L/min) and simulator effort were kept constant when testing each mask against the baseline (background) condition without any mask. The tidal volume, respiratory rate, minute ventilation, and baseline relative humidity inside the chamber (and outside the PPE) were the same across all conditions (*p* > 0.9). Statistical analysis was performed using Generalized Linear Mixed Models (IBM SPSS v25, Armonk, NY).

## Results

Representative raw tracings of particle concentration are shown in Figure 2 for the baseline condition without a mask and for the N95 and N99 masks. Baseline particle counts were 2,935 ± 555 (mean ± standard deviation) cm^−3^, and much higher than that for the N95 mask (1,348 ± 92 cm^−3^) and N99 mask with elastomeric lining (279 ± 164 cm^−3^; *p* <0.0001; Figure 2). The filtering efficiency was 54.1 and 90.5% for the N95 and N99 masks, respectively.

## Discussion

We found that for fine aerosols with diameters of 350 ± 6 nm, the N99 mask with elastomer lining had superior filtration efficiency than the N95 respirator and they both performed lower than their ratings. In general, the total efficiency for filtration by a single-fiber filter includes the summative effects of various mechanisms such as diffusion (for particles smaller than 100 nm); interception (>50 nm), impaction (>100 nm), and settling (>1,000 nm) (6). However, there is a *most penetration* for particles between 80 and 400 nm in diameter (6). Interestingly, such a particle size range is remarkably similar to the particle size of fine aerosols that are most likely to be retained within the respiratory tract (30% retention) and achieve modest deposition in the lower respiratory tract ~10% deposition (100-500 nm). However, these very same particle sizes that correspond to the most penetration particle sizes are more likely to remain suspended in a room as an aerosol and eventually penetrate the mask filter and then be retained in the lung with gradual deposition over multiple breaths. We recognize that there was some variability in the particle count. Sources of concentration variability include perturbations in the system's air flow induced by the lung simulator's respiratory cycles, imperfect mixing, potential variability in particle generation at fixed concentration, and potential particle losses to surfaces. Also, we recognize that while these filtration mechanisms do work on their own, often they work in conjunction with one or more other mechanisms depending on particle size and velocity. For instance, large particles with low velocities are filtered more efficiently *via* interception and gravity, particles >1 μm will filter out with impaction and interception at high velocities, particles between 0.2 and 0.3 μm will filter out with impaction, interception, and diffusion at high velocities, and lastly particles between 0.1 and 1 μm will filter out with diffusion and interception with decreasing velocity as particle size increases.

Others have reported poor field performance of N95 masks, which may be due to gaps between the N95 mask and the mannequin face that could be prevented by elastomeric linings (7). There are two important aspects of the mask that can be manipulated to improve filtration efficiency. The filter rating and the mask lining. We chose to compare N95 masks without elastomeric lining to N99 mask with elastomeric lining because these are generally the types of mask plus lining combinations that were available at the beginning of the pandemic. The N99 mask with elastomeric lining in our study was made by combining readily available air-filters (N99 filter rating) into 3D printed masks with elastomeric linings [that could also be replaced by continuous positive airway pressure (CPAP) masks for sleep apnea treatment (11)] to yield the N99 rating mask with elastomeric lining that was tested in our study. There are over 7 million patients with sleep apnea in the U.S. alone who use continuous positive airway pressure (CPAP) machines and N99 filters used in our study were more readily available than N95 masks at the start of the pandemic (11). Such an issue can again arise in future pandemics when supply chains are disrupted. We recognize that “laboratory-based” filtration efficiency measurements is ideally not a combination of leakage around the mask and the filter efficiency, but when simulating real-world conditions in the laboratory, we found lower than anticipated levels of filtration efficiency due to mask filter leakage and additional mask leakage.

Another explanation for the poor performance of the N95 mask observed in our study could be the cumulative accumulation of retained particles that had penetrated through the filter over the course of each breathing run. Such multiple breath simulation is relevant considering that healthcare workers may be within a patient room for >3 min and accumulate and deposit aerosols in their lungs over multiple breaths that can spread the virus several meters or even the entire room (12). In conclusion, we believe that public health recommendations should encourage masks with greater filtration efficiency with elastomeric linings during respiratory viral outbreaks until inoculation is deemed sufficient.

## Data Availability Statement

The raw data supporting the conclusions of this article will be made available by the authors, without undue reservation.

## Author Contributions

AS, KM, DL, and SP: conception, funding, study design, analysis, drafting the manuscript, and reviewing and editing the manuscript critically. CS, SC, and CM: study design, analysis, conducting experiment, and reviewing and editing the manuscript critically. All authors contributed to the article and approved the submitted version.

## Conflict of Interest

The authors declare that the research was conducted in the absence of any commercial or financial relationships that could be construed as a potential conflict of interest.
